# Autophagy inhibition potentiates the anti-EMT effects of alteronol through TGF-β/Smad3 signaling in melanoma cells

**DOI:** 10.1038/s41419-020-2419-y

**Published:** 2020-04-07

**Authors:** Yong Bao, Zhi Ding, Peng Zhao, Jun Li, Ping Chen, Jie Zheng, Zhongming Qian

**Affiliations:** 1 Department of Pharmacology and Biochemistry, School of Pharmacy, Fudan University, 826 Zhangheng Road, 201203 Shanghai, China; 2 Department of Plastic Surgery, Huashan Hospital, Fudan University, No. 12 Urumchi Middle Road, Jing’an District, 200040 Shanghai, China; 3 Research Center for Medicine and Biology, Zunyi Medical University, No. 6 University west Road, Zunyi, 563000 Guizhou, China

**Keywords:** Skin cancer, Pharmacology

## Abstract

Accumulating evidence demonstrated that alteronol, a novel compound that has a similar structure with paclitaxel, exerts anticancer effects against diversified tumors. However, whether alteronol induces autophagy and the relationship between its anticancer effects and autophagy in melanoma remains elusive. In this study, we show that alteronol induces not only anti-proliferation activity and apoptosis but also autophagy in A375 and UACC62 cells. In addition, alteronol inhibits A375 and UACC62 cells invasion and migration by preventing the epithelial–mesenchymal transition (EMT). Blocking autophagy enhances alteronol-induced apoptosis and anti-EMT effects in vitro and in vivo. Mechanistically, we find that alteronol significantly inhibits Akt/mTOR and TGFβ/Smad3 pathways, and co-treatment with autophagy inhibitor 3-MA further potentiate these effects. Our results suggest that alteronol induces cyto-protective autophagy in melanoma cells through inhibition of Akt/mTOR pathway, thus attenuates apoptosis and promotes melanoma cell EMT through TGF-β/Smad3 pathway. Combination with alteronol and autophagy inhibitor 3-MA may be a potential treatment for melanoma as it not only significantly inhibited tumor growth but also suppressed tumor invasion and migration as anti-metastasis agent.

## Introduction

Melanoma, arising from pigment-producing melanocytes, is the most aggressive malignant skin cancer that accounts for 80–85% of all the skin cancer-related death, with about 100,000 fatalities every year^1–3^. Although current melanoma therapies including surgery, chemotherapy, and biological therapy are available for patients, these treatments are still very limited and frequently induce unwanted side effects, drug resistance, and recurrence. Novel immunotherapy agents such as nivolumab, pembrolizumab, and ipilimumab have greatly improved outcome. However, the prognosis is still poor with the median survival barely at 25.9 months by 2015^4^. Therefore, further development of novel and effective therapeutic agents for malignant melanoma are urgently needed.

**Fig. 1:**
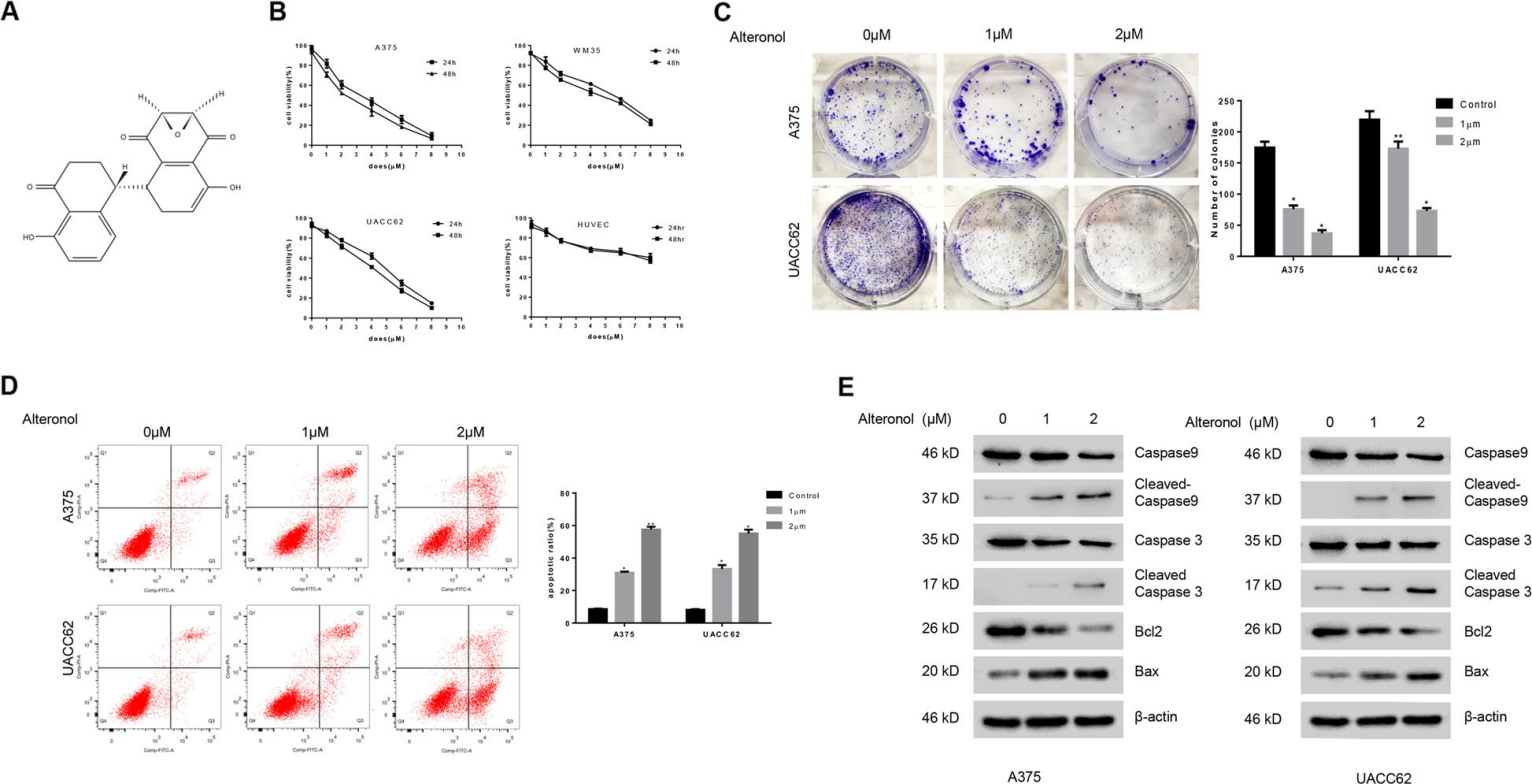
Alteronol inhibits cell viability in melanoma cells. **a** The chemical structure of alteronol. **b** A375, WM35, UACC62, and HUVEC cells were treated with various concentrations (0, 2, 4, 6, 8 μm) of alteronol for 24 and 48 h. The cell viability was evaluated by MTT assay. All data are representative of three independent experiments. **c**–**e** A375 and UACC62 cells were treated with alteronol as indicated. **c** Clone formation assays were performed. **d** The percentage of apoptotic cells were measured by Annexin V/PI staining. **e** Apoptosis-related protein were detected by western blotting. All data are presented as the mean ± S.D. for three independent experiments, *p < 0.05, **p < 0.01.

Paclitaxel is an effective anti-tumor drug isolated from the bark of the yew tree through microbial strain and used as the first-line chemotherapy drug in various types of cancer^5^. Alteronol (Fig. 1a) is a novel compound that has the same source and similar structure with paclitaxel. Previous studies showed that alteronol has anti-tumor effects on several types of cancer, such as gastric cancer, breast cancer, prostate cancer, and melanoma by inducing cell apoptosis, cell cycle arrest, and inhibition of cell invasion/migration^6–10^. However, these underlying mechanisms remain unclear.

Autophagy is an evolutionarily conserved process that directs cytoplasmic proteins and dysfunctional organelles to lysosomes for degradation and recycling^11,12^. Autophagy generally recognized as a protective process to maintain the intracellular homeostasis and facilitated cancer cells survival upon chemotherapeutic agent treatment^13,14^. However, anti-tumor agents, such as etoposide, temozolomide, and everolimus can trigger autophagic cell death to potentiate the cytotoxicity in cancer cells^15,16^. Thus, the role of autophagy seems to be context dependent upon chemotherapy.

The relationship between autophagy and epithelial–mesenchymal transition (EMT) is also controversial. Accumulating evidences revealed that autophagy promote cancer cells migration and invasion upon starvation or hypoxia^17,18^; while others suggested that autophagy could inhibit cancer cells EMT^19^ and metastasis after anti-tumor agents treatment^20^. Therefore, we were interested in evaluation of the anti-tumor effects of alteronol and the relationship between alteronol-induced autophagy and apoptosis or EMT in melanoma cells. Furthermore, we suggested whether targeting autophagy, could potentiate the therapeutic effects of alteronol in the proliferation, apoptosis and cell invasion, and migration.

## Material and methods

### Cell lines and cultures

Human melanoma cell line UACC62 was cultured in RPMI-1640 medium and cell lines A375 and WM35 were cultured in DMEM medium. Human umbilical vein endothelial cells (HUVECs) were maintained in endothelial cell medium (ECM). All the medium contained 10% fetal bovine serum and 1% penicillin/streptomycin. All cell lines were obtained from cell bank of Chinese Academy of Sciences, Shanghai (Shanghai, China). Cells were maintained in 5% CO_2_ at 37 °C and in humidified incubator.

### Chemicals and reagents

Alteronol with 99% purity was obtained from Shantou Strand Bio Technology Co., Ltd. (Shantou, China). DMSO, 3-methyladenine (3-MA), and 2-mercaptoethanol, 3-(4,5-dimethylthiazol-2-yl)-2,5-diphenyl tetrazolium bromide (MTT) were purchased from Sigma ((Shanghai, China). Annexin V-FITC and propidium iodide (PI) was obtained from BD Biosciences (San Jose, USA). Primary antibodies: Caspase3, Caspase9, AKT1, p-AKT1(Ser473), Beclin1, mTOR, p-mTOR(Ser2448), Bcl-2, p62, Bax, Smad3, phospho-Smad3 (Ser423/425), and LC3 were from Cell Signaling Technology (Danvers, MA, USA). The antibodies for β-actin, E-cadherin, and vimentin were from Santa Curz Biotechnology (Santa Cruz, CA).

### Cell viability and colony formation

Cell viability and colony formation assay were performed as described previously^21,22^. Briefly, cells were seeded in 96-well plates (5000 cells/well) and then were subjected to various indicated treatments. Cells viability were measured by MTT, and the absorbance at 570 nm was quantified. Results are expressed as mean ± SD from three technical replicates. For colony formation assay, cells were seeded in triplicate into six-well plate (~200 cells/well) and incubated with increased concentrations of alteronol for 14 days. After staining with crystal violet (0.1%), the colonies were visualized and quantified.

### Apoptosis analysis

Cell apoptosis was measured using an Annexin V-FITC/PE Apoptosis kit (BD Biosciences, San Jose, USA) according to the manufacturer’s instructions^22^. Briefly, cells were plated into a six-well plate (5 × 10^4^ cells/well) for 24 h and then subjected to various indicated treatments. Cells were then harvested and washed with PBS and were incubated with annexin V-binding buffer and 1 mg/ml PI for 10 min at room temperature. After incubation, cells were measured by flow cytometry and data analysis was measured by FlowJo (BD Biosciences).

### Western blot analysis

Treated and un-treated cells were collected and lysed in RAPI lysis buffer. After quantification by using BCA reagent, proteins were separated on SDS–PAGE and transferred to nitrocellulose membrane. Then, membranes were blocked in 5% non-fat milk and incubated with primary antibodies at 4 °C overnight. The membranes were then washed and incubated with secondary antibody at room temperature for 1 h. Blots were visualized by using Millipore’s enhanced chemiluminescence detection system (ChemiDoc Touch, BioRad).

### CRISPR/Cas9 knockout (KO) system and mCherry-EGFP-LC3 stable cell line

Beclin1 KO cell lines were generated by using a lentiCRISPRv2-Beclin1 plasmid (5′-CACCGATCTGCGAGAGACACCATCC-3′) purchased from Addgene (#99574). mCherry-EGFP-LC3 stable cell line were generated by using a mCherry-EGFP-LC3 plasmid purchased from Addgene (#22418). CRISPR/Cas9 KO system and mCherry-EGFP-LC3 stable cell line were performed as described previously^21^. The recombinant lentivirus encoding mCherry-EGFP-LC3 or lentiCRISPRv2-Beclin1 produced by 293 T cells were used to transduce A375 or UACC62 cells. The stable cell lines were established by selection with 2 μg/ml of puromycin (Invitrogen).

### Evaluation of fluorescent mCherry-EGFP-LC3 puncta

Autophagy was measured by quantifying the number of yellow or red puncta in mCherry-EGFP-LC3 stable cell lines^21^ treated with or without alteronol. Images acquisition was performed by using LSM710 confocal microscope with a ×63 oil objective (Carl Zeiss).

### Transmission electron microscopy (TEM)

TEM assay was performed as described previously^23^. Briefly, alteronol-treated A375 cells were harvested and fixed in 0.1 M sodium cacodylate buffer containing 2.5% glutaraldehyde at 4 °C and post-fixed with 1% osmium tetroxide for 3 h. The sample was dehydrated and embedded in EMbed 812 (Electron Microscopy Sciences). Ultrathin sections (60 nm) were prepared and stained with uranyl acetate for 3 min and then with lead citrate, and examined on Philips CM-12 electron microscope (FEI).

### Cell migration and invasion assay

Cell migration or invasion was measured by using Cultrex® 96 Well Cell Migration Assay or Cultrex® 96 Well BME Cell Invasion Assay (Trevigen, USA) according to a standard protocol^21^. Briefly, cells (5 × 10^4^ cells/well) were trypsinized and seed on the upper chamber of a transwell (migration) or a 1xBME solution-coated transwell (invasion) in serum-free media. The bottom chamber contained DMEM or RPMI 1640 media with 10% FBS and alteronol or DMSO with or without 3-MA as indicated. After 24 h of incubation at 37 °C, cells were labeled with 5 μg/mL calcein-AM in DMEM at 37 °C for 1 h; and counted under a fluorescence microscope. Results are expressed as mean ± SD from three technical replicates.

### Immunofluorescence microscopy

A375 and UACC62 cells were plated into coverslips (5000 cells/well) for 24 h and then subjected to various indicated treatments. Immunofluorescence confocal microscopy was performed as described^21^. Briefly, cells were fixed and permeabilized with Triton X-100 for 10 min on ice. After washing with PBS for three times, cells were incubated with anti-E-cadherin (#14472, Cell Signaling Technology) or anti-vimentin (#5741, Cell Signaling Technology) antibodies for 1 h at room temperature. Cells were washed with PBS, and incubated with Alexa Fluor secondary antibodies (Invitrogen) for 1 h. DAPI (Vectashield, H1200) was used to stain nuclei. Images were acquired with an LSM510 confocal microscope with a ×63 objective (Carl Zeiss).

### Animal xenograft evaluation

All animal procedures were conducted in accordance with the protocols approved by the Institutional Animal Care and Use Committee at Fudan University. Xenograft tumor models were established as described previously^24^. Briefly, 6 weeks old immunocompromised BALB/c male mice were obtained from the Shanghai Laboratory Animal Center (Shanghai, China). A375 tumors were induced into them by injecting subcutaneously into the hind flank with 5 × 10^6^ cells suspended in 100 μl RPMI 1640. When tumors reached a size of ~80 mm^3^, mice were randomly assigned into four groups (*n* = 5/group) and intraperitoneal injections of saline (vehicle control), or alteronol (3 mg/kg/d) with/without 3-MA (25 mg/kg/d). Mice were killed 28 days after A375 cells inoculation, and xenograft tumors were weighed and used for western blotting.

### Statistical analyses

All the experiments were performed three times independently. The data were presented as the mean ± SD and analyzed by using one-way ANOVA or two-sample equal variance Student’s *t*-test. Statistical analysis was measured by using Graphpad Prism software. *P* < 0.05 was considered as significant.

## Results

### Alteronol inhibits cell proliferation in melanoma cells

We first investigated whether alteronol has cytotoxic effect on human melanoma cells by using MTT assays. Melanoma cell lines A375, UACC62, and WM35, and benign cell line HUVEC were treated with different concentrations (0, 1, 2, 4, 6 and 8 μm) of alteronol for 24 and 48 h, respectively (Fig. 1b). The results showed that alteronol significantly inhibited melanoma cell growth in a dose-dependent and time-dependent manner. The IC_50_ values of all these cell lines are listed in Table 1. Alteronol had most potent cytotoxic effect on A375 cells as compared with other melanoma cell lines. However, the benign cell line HUVEC was only weakly affected by alteronol. These results indicated a preference of alteronol in killing melanoma cells over normal cells. We further measured the long-term effect of alteronol by performing the colony formation assay, and showed that the colonies were significantly decreased after alteronol treatment in both UACC62 and A375 cells (Fig. 1c).

**Table 1:** Cell viability of alteronol in melanoma cells.

Cell lines	IC50 (μM)	
	24 h	48 h
A375	3.72 ± 0.32	2.53 ± 0.43
WM35	5.31 ± 0.21	4.42 ± 0.27
UACC62	4.92 ± 0.60	4.47 ± 0.16
HUVEC	>30	>30

Melanoma cells were treated with increasing concentrations of alteronol for 24 and 48 h, respectively. The cell growth was evaluated by MTT assay. Experiments were performed in triplicate, and the results were presented as the mean ± SD.

### Alteronol induces apoptosis in melanoma cells

To investigate whether alteronol induced melanoma cell death through apoptosis, we analyzed the apoptotic effects on A375 and UACC62 cells by using flow cytometry. After treatment with increased concentrations of alteronol, the percentage of apoptotic cells were elevated from 8.57% (in control group) to 57.59% (2 μm Alteronol) in A375 cells and from 8.15% (in control group) to 51.17% (2 μm Alteronol) in UACC62 cells (Fig. 1d). We further examined the apoptosis-related proteins in alteronol-treated cells. Consistent with flow cytometry analysis, the protein level of cleaved caspase 3, caspase 9, and Bax were all increased after alteronol treatment while the level of Bcl2 was decreased (Fig. 1e). These results demonstrated that alteronol could induce caspase-dependent apoptosis in human melanoma cells.

### Alteronol induces autophagy in melanoma cells

**Fig. 2:**
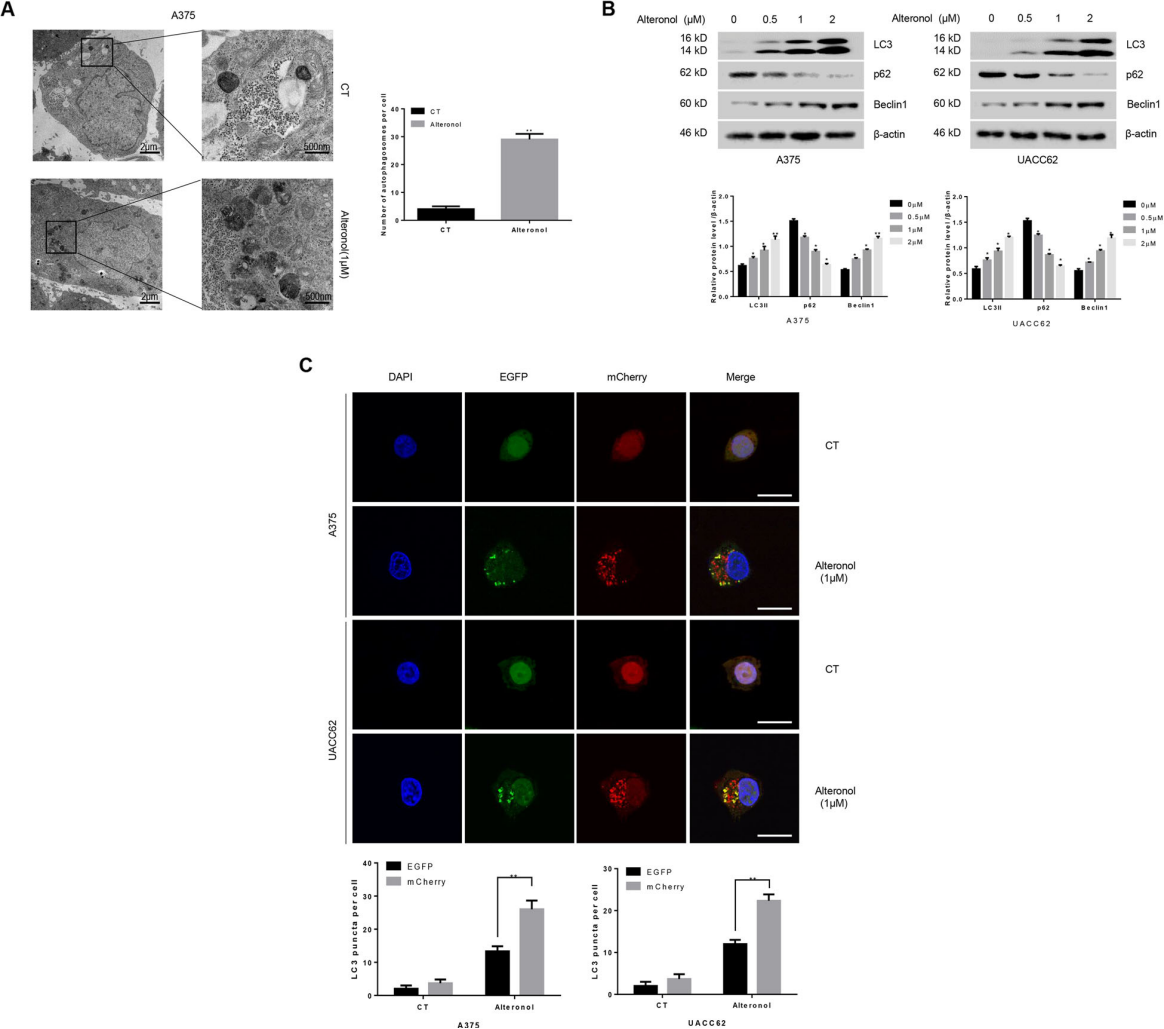
Alteronol induces autophagy in melanoma cells. **a** TEM analysis of autophagy after A375 cells were treated with or without alteronol. Ten cross-sections were counted in each experiment. **b** A375 and UACC62 cells were treated with indicated concentrations of alteronol and autophagy-related proteins were detected. **c** A375 and UACC62 cells transfected with mCherry-EGFP-LC3 were treated with or without 1 μM alteronol for 24 h and the statistics of red or yellow puncta per cell calculated from 10 different images were illustrated. Sclar bar: 10 μm. All data are presented as the mean ± S.D. for three independent experiments, *p < 0.05, **p < 0.01.

To investigate whether alteronol induces autophagy in melanoma cells, TEM, the gold standard for detecting autophagy was performed. As shown in Fig. 2a, double membrane or multi-membrane structures were accumulated in A375 cells after treatment with alteronol. Moreover, the accumulation of LC3II and the degradation of p62 as well as the expression of Beclin1 were all dose-dependently increased after alteronol treatment (Fig. 2b). To further investigate the effect of alteronol on the autophagic process, A375 and UACC62 cells were stably transfected with the mCherry-EGFP-LC3 plasmids, which the yellow puncta (mCherry-EGFP-positive) represent autophagsomes and red puncta (mCherry-positive) represent autolysosomes whose EGFP fluorescence was quenched in acidic pH solution^25^. After incubating with 1 μM alteronol for 24 h, we observed that both autophagsomes (yellow puncta) and autolysosomes (red puncta) accumulated in A375 and UACC62 cells. Importantly, the number of autolysosomes (red puncta) increased more rapidly than autophagosomes (yellow puncta) which indicated that alteronol activated complete autophagic flux in melanoma cells (Fig. 2c).

### Alteronol induces autophagy by targating Akt/mTOR pathway

**Fig. 3:**
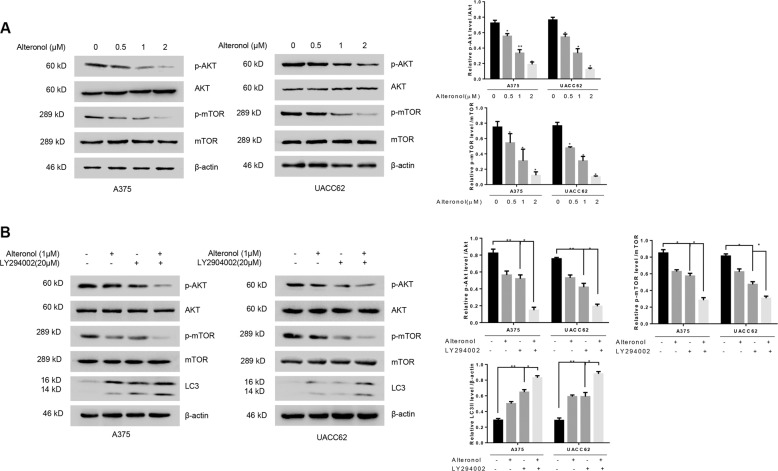
Alteronol induces autophagy through Akt/mTOR pathway. **a** A375 and UACC62 cells treated with increased concentrations (0, 0.5, 1, 2 μM) of alteronol. Immunoblotting was performed with antibodies as indicated. **b** A375 and UACC62 cells treated with/without alteronol in the presence or absence of LY294002 as indicated. Immunoblotting assays were performed to measure the phosphorylation status of Akt and mTOR and the accumulation of LC3. All data are presented as the mean ± S.D. for three independent experiments, *p < 0.05, **p < 0.01.

Akt/mTOR pathway is one of the major regulators in autophagy^26^, we therefore examined whether Akt/mTOR pathway is required for the alteronol-induced autophagy. After incubated with increasing concentrations (0, 0.5, 1 and 2 μm) of alteronol for 24 h, A375 and UACC62 cells were harvested for immunoblotting assay. The results showed that the phosphorylation level of Akt and mTOR were does-dependently decreased after alteronol treatment (Fig. 3a). Moreover, co-treatment with alteronol and LY294002, an inhibitor of the Akt/mTOR-signaling pathway, greatly promote the accumulation of LC3II as compared with alteronol treatment alone (Fig. 3b). There results suggested that Akt/mTOR pathway is required for alteronol-induced autophagy in A375 and UACC62 cells.

### Alteronol-induced autophagy attenuates apoptosis

**Fig. 4:**
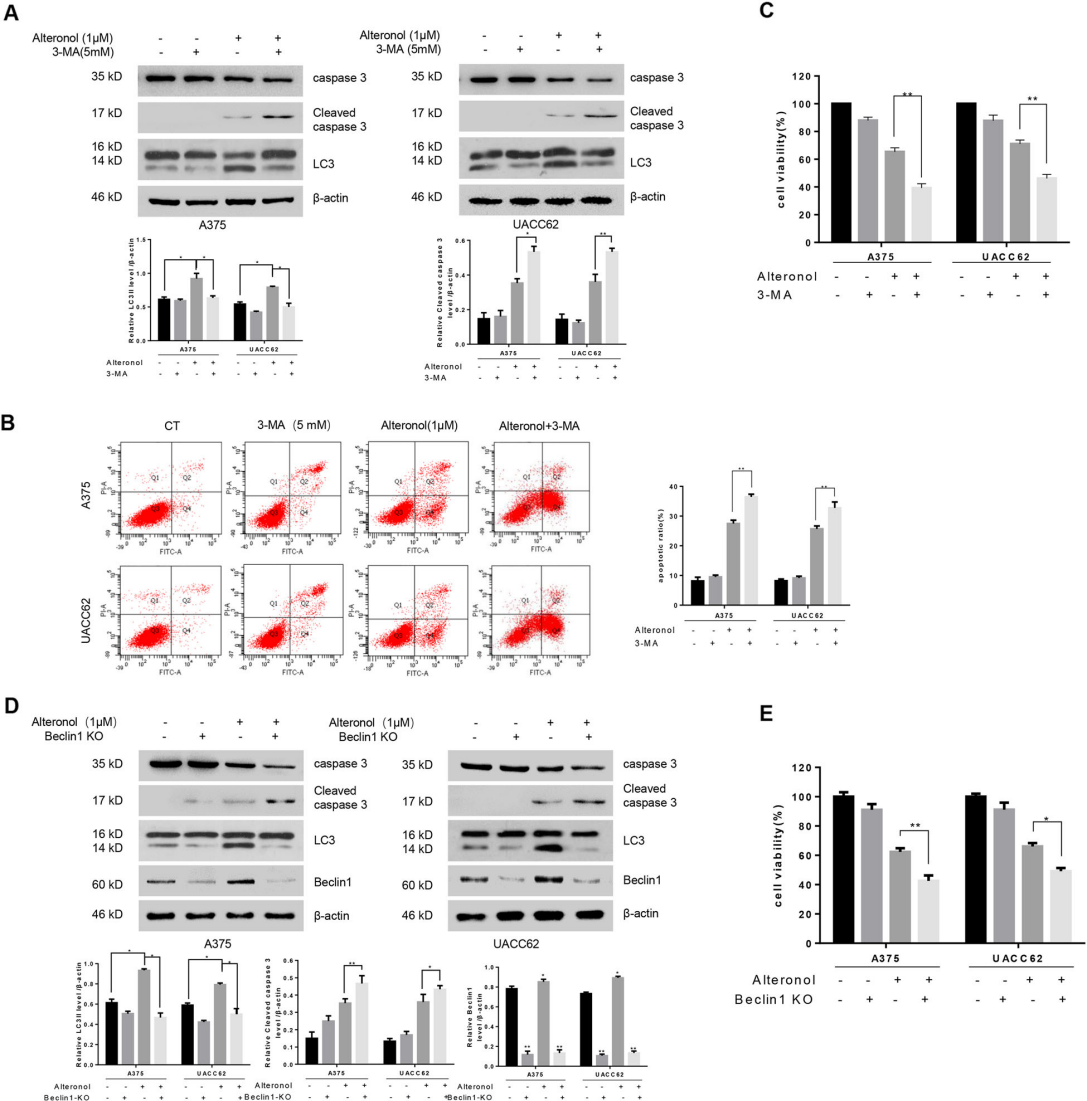
Alteronol-induced autophagy attenuates apoptosis. **a**–**c** A375 and UACC62 cells were treated with or without alteronol in the presence or absence of 3-MA. **a** caspase 3 and LC3 were measured by immunoblotting. **b** The percentage of apoptotic cell was evaluated by flow cytometry. **c** The cell viability was evaluated by MTT. All the data were expressed as the mean ± S.D. of triplicate experiments. *p < 0.05, **p < 0.01. **d**, **e** A375 and UACC62 cells with or without Beclin1 knockout in the presence or absence of alteronolt as indicated. **d** caspase 3 and LC3 were analyzed by immunoblotting. **e** The cell viability was evaluated by MTT. The data were expressed as the mean ± S.D. of triplicate experiments. *p < 0.05, **p < 0.01.

To further investigate alteronol-induced autophagy, 3-MA, an autophagy inhibitor, was applied. As shown in Fig. 4a, 3-MA significantly inhibited alteronol-induced autophagy, as evidenced by the down-regulation of LC3II expression in A375 and UACC62 cells. Moreover, 3-MA could augment alteronol-induced cleavage of caspase3 apoptotic effects in A375 and UACC62 cells (Fig. 4b). We further examined this combination effect by using MTT assays, and the results showed that the cell viability were dramatically reduced in A375 and UACC62 cells co-treated with alteronol and 3-MA as compared with alteronol treatment alone (Fig. 4c). These data suggested that alteronol might induce a cyto-protective autophagy in A375 and UACC62 cells. We further KO Beclin1 to block autophagy in A375 and UACC62 cells, and found that Beclin1 KO effectively increased alteronol-induced apoptosis (Fig. 4d) and further suppressed the cell viability (Fig. 4e). Taken together, these results suggested that inhibition of autophagy potentiated alteronol-induced apoptosis in human melanoma cells.

### Inhibition of autophagy potentiated the anti-EMT properties of alteronol

**Fig. 5:**
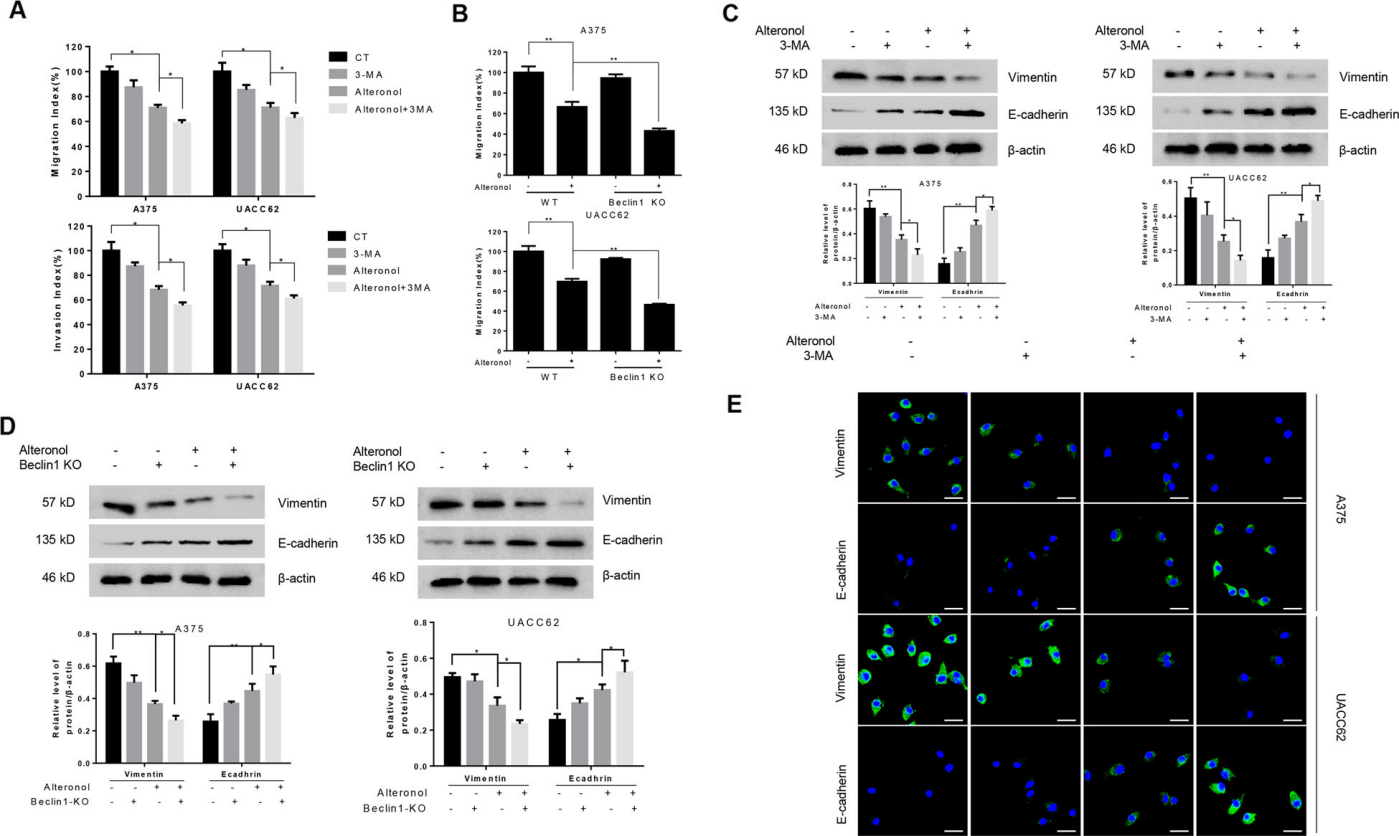
Inhibition of autophagy potentiated the anti-EMT properties of alteronol. **a**, **c** and **e** A375 and UACC62 cells were treated with or without alteronol in the presence or absence of 3-MA. **a** Transwell migration or invasion assays were preformed. Scale bar: 10 μm. The data were expressed as the mean ± S.D. of triplicate experiments. *p < 0.05. **c** Western blot analysis of EMT-related proteins. **e** Immunofluorescence of the E-cadherin and vimentin. **b**, **d** A375 and UACC62 cells with or without Beclin1 knockout in the presence or absence of alteronol as indicated. **b** Transwell migration assays were preformed and the data were expressed as the mean ± S.D. of triplicate experiments. **p < 0.01. **d** Western blot analysis of EMT-related proteins.

Besides direct antitumor effect, we also investigated the role of alteronol-induced autophagy on melanoma cells metastasis by performing cell invasion and migration assays. Alteronol significantly reduce the invasion and migration rate in A375 and UACC62 cells, while 3-MA augmented this effect (Fig. 5a). In addition, alteronol treatment enhanced the reduction of cell migration rate in Beclin1 KO A375 and UACC62 cells as compared with Beclin1 WT (Fig. 5b). These results suggested that alteronol-induced cyto-protective autophagy might promote melanoma cell invasion and migration.

To investigate whether this autophagy promoted cell invasion and migration associated with EMT, we measured the epithelial and mesenchymal cell markers in A375 and UACC62 cells. Alteronol treatment could increase the protein level of E-cadherin (epithelial markers), while decreased the protein level of vimentin (mesenchymal marker), and in combination with 3-MA or Beclin1 KO could further enhance this effect (Fig. 5c, d). Consistent with the result by western blotting, immunofluorescence showed that combination of alteronol with 3-MA further increased the expression level of E-cadherin and reduced the expression level of vimentin (Fig. 5e). Together, these results demonstrated that alteronol-induced autophagy could promote melanoma cells invasion and migration. Inhibition of this autophagy could further potentiate this anti-EMT function of alteronol.

### TGF-β/Smad3 signaling is involved in autophagy-induced EMT

**Fig. 6:**
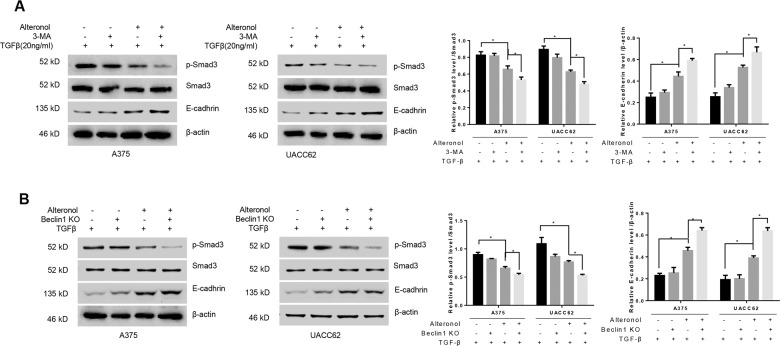
TGF-β/Smad3 signaling is involved in autophagy-induced EMT. **a** Western blot assays were measured in A375 and UACC62 cells treated with TGF-β and/or 3-MA with or without alteronol as indicated. **b** Western blot assays were measured in A375 and UACC62 cells treated with TGF-β and/or alteronol in the presence or absence of Beclin1 knockout as indicated. All data are presented as the mean ± SD for three independent experiments, *p < 0.05, **p < 0.01.

TGFβ/Smad3 is an important signal pathway that mediate EMT^27^ and many studies have confirmed that autophagy can induce TGFβ/Smad3 activation^28^. Therefore we investigated whether this alteronol-induced autophagy promote EMT through TGFβ/Smad3 pathway. As the basal protein level of TGFβ and p-Smad3 were not highly expressed in A375 and UACC62 cells, we treated these cells with TGFβ1 (20 ng/ml) cytokine and found the up-regulation of p-Smad3 and down-regulation of E-cadherin (Supplementary Fig. S1). Alteronol treatment could significantly reduce this TGFβ1-induced up-regulation of p-Smad3 and down-regulation of E-cadherin. Moreover, autophagy inhibition with 3-MA or Beclin1 KO further potentiate the inhibition of TGFβ–Smad3 pathway (Fig. 6a, b). Together, these results showed that blocking autophagy could enhance anti-EMT function of alteronol through TGF-β/p-Smad3 pathway in A375 and UACC62 cells.

### Co-treatment with alteronol and 3-MA potentiated anti-tumor activities in vivo

**Fig. 7:**
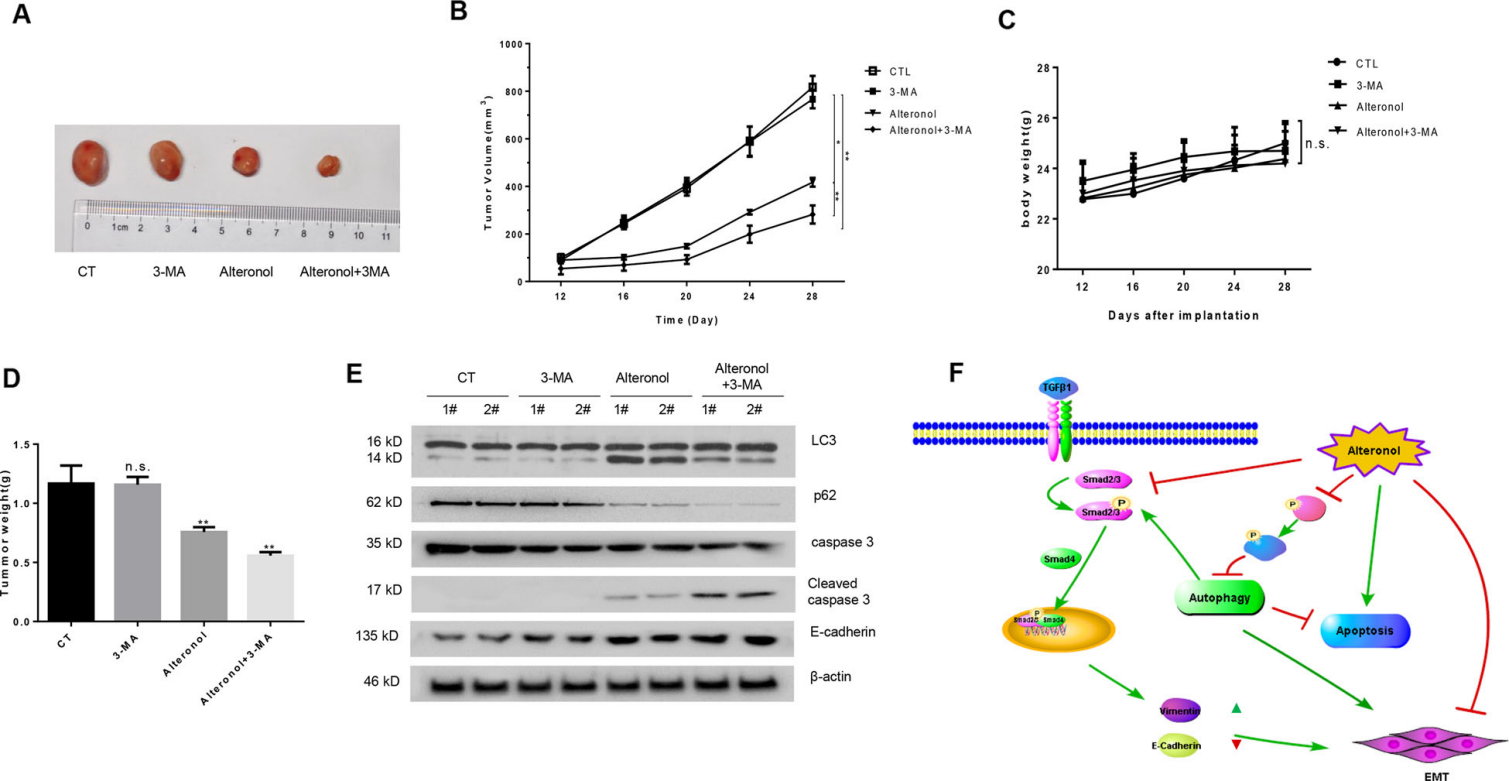
Co-treatment with alteronol and 3-MA potentiated anti-tumor activities in vivo. Mice with A375 xenografts were randomly divided into four groups and intraperitoneal injections of saline (vehicle control) or alteronol (3 mg/kg) with/without 3-MA (25 mg/kg) (*n* = 5) were given. **a** Photographs of tumors removed from xenografted nude mice in each treatment group. **b**, **d** Tumor volumes and tumor weight from each treatment group during the study period. **c** Mice body weight curves from each treatment group. **e** Western blot assays were performed in the xenograft tumor tissues from two different mice in each treatment group. **f** Schematic diagram of the mechanism of alteronol-induced autophagy in melanoma cells. All data are presented as the mean ± SD (*n* = 5). n.s. = no significant, *p < 0.05, **p < 0.01.

After taking into account the anti-tumor effects of alteronol in vitro, we subsequently examined these effects in a xenograft model. The mice were injected with A375 cells to generate xenograft tumors and then intraperitoneal injections of saline (vehicle control) or alteronol (3 mg/kg/d) with/without 3-MA (25 mg/kg/d) were given. Consistent with the in vitro results, combination treatment elicited a strong antitumor effect as compared with 3-MA or alteronol treatment alone (Fig. 7a). In addition, no significant adverse effects were observed in all these groups, indicating that co-treatment with 3-MA significantly enhanced alteronol-induced anti-tumor effects in vivo (Fig. 7b–d).

Next, we investigated the alteronol-induced apoptosis, autophagy, and EMT in xenograft tumors. Cleaved caspase-3, LC3-II, and E-cadherin protein expression levels were all increased after alteronol treatment. In addition, autophagy inhibitor 3-MA could further potentiate these effects (Fig. 7e). These results suggested that alteronol inhibits A375 xenograft growth and migration/invasion, and inhibition of autophagy potentiated the inhibitory effects of alteronol.

## Discussion

Alteronol is a new type of compound that has a similar structure with the first-line chemotherapy drug paclitaxel. It has been reported that alteronol could inhibit melanoma cells B16F10 and B16F1 invasion and migration^15^, while the underlying mechanism remains unknown. In this study, we showed that alteronol induced caspase-dependent apoptosis and cyto-protective autophagy in melanoma cells. In addition, alteronol inhibited melanoma cell invasion and migration by preventing the EMT. Blocking autophagy enhanced the cytotoxicity and anti-EMT effect of alteronol in a synergistic manner (Fig. 7f).

Autophagy plays an important role in cancer therapy. However, whether alteronol could induce autophagy in melanoma cells is unclear. Our results for the first time demonstrated that alteronol could induce autophagy in melanoma cells. Further research by using mCherry-EGFP-LC3 plasmids found that alteronol also activated the complete autophagic flux. Many nature products could induce autophagy through the inhibition of Akt/mTOR pathway^29^, while paclitaxel has been reported to upregulate Akt pathways to compromise its antitumor activity^30^. Our current data showed that alteronol could induce autophagy by down-regulation of the phosphorylation level of mTOR and Akt. LY294002, an inhibitor of the PI3K/Akt/mTOR signaling pathway, could further increase the accumulation of LC3II in alteronol-treated A375 and UACC62 cells. The relationship between autophagy and apoptosis is complicated. Therefore, it is essential to identify whether autophagy could promote or prevent apoptosis. We found that alteronol-induced autophagy tends to play a cyto-protective role and may be associated with drug resistance in melanoma cells. Inhibition of autophagy by using autophagy inhibitor 3-MA or KO Beclin1 could dramatically enhance alteronol-induced apoptosis as compared with alteronol alone in vitro and in vivo.

The relationship between autophagy and EMT is also complicated. Starvation or hypoxia-induced autophagy is critical for cancer cell migration and invasion^17,18^; while some nature products like tetrandrine could induce autophagy to block cancer cells EMT and metastasis^20^. In our studies, we showed that alteronol effectively inhibits melanoma cells EMT as the upregulation of E-cadherin and down-regulation of vimentin. In addition, alteronol could reduce the phosphorylation level of Smad3, thus inhibits the TGFβ/smad3 signal pathway. However, we found that alteronol-induced cyto-protective autophagy could promote melanoma cells EMT. Inhibition of this autophagy by using 3-MA or Beclin1 KO further potentiated the anti-EMT effects of alteronol in a synergistic manner. Notably, we found that alteronol could also inhibit E-cadherin protein level and cell migration/invasion without adding TGFβ (Fig. 5), which indicates that there might be other pathway involved in the alteronol-mediated inhibition of EMT in melanoma cells. In this respect, transcriptome sequencing will be needed to fully understand this regulation process.

In conclusion, our results demonstrated that alteronol is an effective anti-melanoma agent both in vitro and in vivo, and the combination of alteronol and autophagy inhibitor 3-MA may have multiple beneficial effects as a potential treatment for melanoma.

## Supplementary information

Supplementary figure legends

Supplementary figure 1
