# Supplementary material for: Autophagy inhibition potentiates the anti-EMT effects of alteronol through TGF-β/Smad3 signaling in melanoma cells
Source: Cell Death Dis. 2020 Apr 7;11(4):223. doi: 10.1038/s41419-020-2419-y (PMC7138813; doi:10.1038/s41419-020-2419-y)
Supplement: Supplementary file 1 — Supplementary figure legends [file 41419_2020_2419_MOESM1_ESM.docx]

## Supplementary Information

**Supplementary Figure Legends**

**Fig. S1** TGFβ1 up-regulation p-Smad3 and EMT in A375 and UACC62 cells.

A and B A375 and UACC62 cells treated with or without TGF-β1 and immunoblotting or cell migration assays were performed.
