# Supplementary figures and images for: Autophagy inhibition potentiates the anti-EMT effects of alteronol through TGF-β/Smad3 signaling in melanoma cells
Source: Cell Death Dis. 2020 Apr 7;11(4):223. doi: 10.1038/s41419-020-2419-y (PMC7138813; doi:10.1038/s41419-020-2419-y)

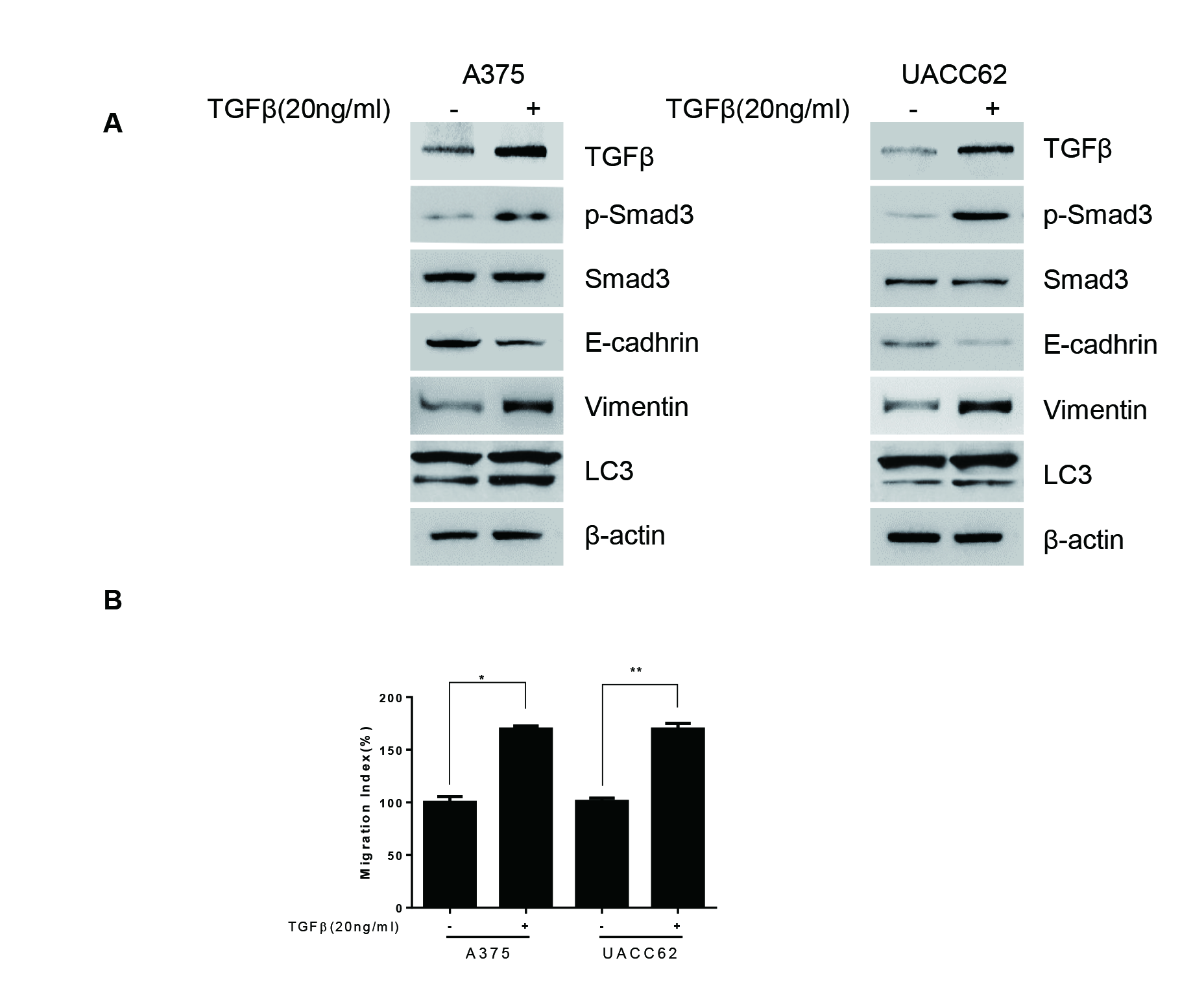

Supplement: Supplementary file 2 — Supplementary figure 1 [file 41419_2020_2419_MOESM2_ESM.tif]
